# A phase II clinical trial to investigate the effect of pioglitazone on ^18^F-FDG uptake in malignant lesions

**DOI:** 10.1186/s13550-015-0128-9

**Published:** 2015-09-25

**Authors:** Yeon-Hee Han, Seong Young Kwon, Jeonghun Kim, Chang Ju Na, Sehun Choi, Jung-Joon Min, Hee-Seung Bom, Young-Chul Kim, In-Jae Oh, Han-Jung Chae, Seok Tae Lim, Myung-Hee Sohn, Hwan-Jeong Jeong

**Affiliations:** Department of Nuclear Medicine, Research Institute of Clinical Medicine of Chonbuk National University-Biomedical Research Institute of Chonbuk National University Hospital, Cyclotron Research Center, Molecular Imaging and Therapeutic Medicine Research Center, Chonbuk National University Medical School and Hospital, Jeonju, Jeonbuk Republic of Korea; Department of Nuclear Medicine, Chonnam National University Hwasun Hospital, Hwasun-gun, Jeonnam Republic of Korea; Lung and Esophageal Cancer Clinic, Chonnam National University Hwasun Hospital, Hwasun-gun, Jeonnam Republic of Korea; Department of Pharmacology, Chonbuk National University Medical School, Jeonju, Jeonbuk Republic of Korea

**Keywords:** PPAR-γ pioglitazone, ^18^F-FDG uptake, Malignant lesion, Inflammatory lesion

## Abstract

**Background:**

We found that ^18^F-2-fluoro-2-deoxy-D-glucose (^18^F-FDG) uptake in malignant lesion was enhanced, and it was decreased in the inflammatory lesion after the use of peroxisome proliferator activated receptor-γ (PPAR-γ) agonist in our previous preclinical study. The purpose of this study was to investigate the effect of PPAR-γ agonist on malignant lesions in clinical ^18^F-FDG positron emission tomography/computed tomography (PET/CT) imaging.

**Methods:**

Forty-three patients were enrolled in this prospective study. We received the approval for the investigator-initiated trials for a phase II human clinical trial from the Korean Food and Drug Administration. On the first day, ^18^F-FDG PET/CT images were acquired from patients without administration of pioglitazone (PIO), which is a PPAR-γ agonist. On the next day, ^18^F-FDG PET/CT images were acquired once again from the same patients after administration of PIO. We measured the ^18^F-FDG uptake in malignant lesions or inflammatory lesions from two ^18^F-FDG PET/CT images. Four different PET parameters were used to compare between the two studies: SUV_max_, SUV_mean_, average activity over 30 % of the isocontour (isocontour, Bq/mL), and isocontour-mediastinal activity (Bq/mL). Additionally, we classified the patients into two groups: the responder or non-responder group according to the presence of PIO effect on skeletal muscle. Furthermore, PET parameters of malignant lesions were analyzed based on the type of malignancy and were compared with those of inflammatory lesions.

**Results:**

All four PET parameters of malignant lesions in the responder group showed increasing patterns after the use of PIO. In the subgroup analysis, the similar pattern was observed in gastrointestinal cancer. In hepatobiliary and pancreatic cancer, SUV_mean_ and isocontour showed statistically significant increase in the presence of PIO. On the other hand, in the non-responder group, all four PET parameters showed decreasing patterns in both malignant and inflammatory lesions after the use of PIO. There was no statistically significant difference in PET parameters of malignant lesions in the non-responder group.

**Conclusions:**

In this study, we found that PIO had the potential to increase ^18^F-FDG uptake of malignant lesions in the patients who showed PIO effect on skeletal muscle. Contrary to our preclinical studies, clinical results had limitations to evaluate malignant lesions in non-responder group. Further larger-scale studies are necessary to elucidate the potential role of PIO on ^18^F-FDG uptake in malignant or inflammatory lesions.

**Trial registration:**

The test for safety and effectiveness of the new efficacy of Pioglitazone to diagnose the malignant tumor and inflammation in F-18 FDG positron emission tomography (PET) study, 12029

## Background

Peroxisome proliferator activated receptors (PPARs) are members of the ligand-activated nuclear receptor superfamily which initiate transcription of an array of genes that are involved in energy homeostasis [1, 2]. So far, three major types of PPARs have been identified and each receptor controls transcription by binding to specific DNA elements: PPAR-α, associated with lipid metabolism; PPAR-β/δ, associated with energy homeostasis; and PPAR-γ, associated with glucose metabolism [3–5]. Transcriptional regulation by PPARs requires heterodimerization with the retinoid X receptor (RXR), and the dimer binds to specific DNA sequence elements called a peroxisome proliferator response element (PPRE) in the promoter region of target genes [6–9].

A wide variety of natural or synthetic compounds were identified as PPAR ligands [6]. Among the synthetic ligands that selectively activate PPAR-γ, thiazolidinedione is an insulin sensitizer that is used to treat hyperglycemia in type 2 diabetes [6, 10]. It reduces insulin resistance in adipose tissue, muscle, and liver [11–14]. Due to improvement in insulin sensitivity, glucogenesis in the liver is decreased and glucose uptake in the peripheral tissues is increased. Currently, two types of thiazolidinediones are commercially available in the market, namely troglitazone and pioglitazone (PIO) [15, 16].

Recently, many researches have demonstrated the anti-inflammatory effects of PPAR receptor [17, 18]. PPAR-γ receptor is expressed in many cell types both within and outside the immune system. Synthetic PPAR-γ agonists including pioglitazone repress the production of inflammatory cytokines in immune-related cells such as CD4+ T cells and monocytes [19]. These reports suggest that PPAR-γ agonists have anti-inflammatory function.

^18^F-2-fluoro-2-deoxy-D-glucose (^18^F-FDG) is a radiopharmaceutical analog of glucose that is used for diagnosis of cancer with increased glucose metabolism. However, ^18^F-FDG uptake is not only found in malignant lesions but also in inflammatory lesions [20–23]. Therefore, inflammatory lesions are likely to be mistaken as malignant lesions owing to intense accumulation of ^18^F-FDG. Because glucose is the primary metabolic substrate of macrophages, immune reaction requires glucose import, primarily through the glucose transporter (Glut) [24].

The effects of PPAR-γ agonist on the peripheral tissue made us curious about how they act on malignant lesions. Other function of PPAR-γ agonists that cause suppression of immune related cells suggests that they may have potential effects on inflammatory lesions. These concepts made us investigate the pharmacological effects of PPAR-γ agonists in preclinical research. In our previous cell and animal studies, we found that rosiglitazone induced Glut 1 in various malignant cells but not in inflammatory cells [25]. Furthermore, in ^18^F-FDG PET/CT images of mouse models, increased ^18^F-FDG uptake was observed in malignant models but not in inflammatory models after administration of PIO [26].

To the best of our knowledge, these effects of PPAR-γ agonists have not been evaluated in human ^18^F-FDG PET/CT images so far. The purpose of this study is to evaluate the effect of PIO on ^18^F-FDG uptake in malignant or inflammatory lesions in clinical ^18^F-FDG PET/CT imaging.

## Methods

### Patients

This was a prospectively designed study. From December 2012 to July 2013, patients who were diagnosed or suspected to have malignant or inflammatory lesions were enrolled at Chonbuk National University Hospital. Exclusion criteria for patients were hypersensitivity to PIO, insulin-dependent diabetes mellitus, diabetic ketoacidosis, active liver disease, or alanine aminotransferase >2.5 times of the upper normal limit, heart failure, and renal insufficiency. Finally 43 patients (30 men and 13 women, mean age, 64.0 ± 11.8 years) were enrolled in this study. Laboratory examinations were performed before and after administration of PIO, and the results were compared for toxicity assessment data. They included serum alanine aminotransferase, aspartate aminotransferase, total bilirubin, direct bilirubin, blood urea nitrogen, creatinine, uric acid, cholesterol, and lactate dehydrogenase. We received the approval for the investigator-initiated trials for a phase II human clinical trial from the Korean Food and Drug Administration. This prospective study was approved by the Institutional Review Board. All patients fully understood the purpose of this study and gave written informed consent.

### ^18^F-FDG PET/CT protocol

On the first day, ^18^F-FDG PET/CT images were acquired from patients in the absence of PIO. All patients fasted for at least 6 h prior to the intravenous injection of ^18^F-FDG, and blood glucose levels in all patients were found to be below 126 mg/dL. Approximately 5.5 MBq of ^18^F-FDG per kilogram of body weight was administered intravenously. Scanning was performed 60 min after ^18^F-FDG administration. Images were obtained from the base of the skull to the proximal thigh by using either a Biograph TruePoint 40 PET/CT scanner (Siemens Medical Solutions, Knoxville, TN, USA) or a Biograph 16 PET/CT scanner (Siemens Medical Solutions, Knoxville, TN, USA). All patients were placed in the supine position with their both arms raised. A low-dose CT scan was obtained first for attenuation correction by using a continuous spiral technique (120 kVp, 45 mA, 0.5 s rotation time). A PET scan was then acquired in a 3-dimensional mode for 2–3 min per bed position. After the acquisition of PET data, the patient underwent a diagnostic CT scan with intravenous contrast (120 kVp, 200 mA, 0.5 s rotation time). The obtained PET data were reconstructed iteratively using an ordered-subset expectation maximization algorithm (128 × 128 matrix, 3.27 mm slice thickness, subset: 21, iterations 2). The next day, patients took PIO orally 2 h prior to ^18^F-FDG PET/CT acquisition because time of peak plasma concentration of PIO is about 2 h. PET/CT images were obtained once again by the same ^18^F-FDG PET/CT protocol without contrast media. ^18^F-FDG PET/CT images of each patient were acquired by using the same PET/CT scanner.

### ^18^F-FDG PET/CT image analysis

18 F-FDG uptake in malignant or inflammatory lesions was measured from two 18 F-FDG PET/CT images. Malignant lesions were selected up to three in a patient. Because some patients had lots of malignant lesions, we limited the number of lesions in a patient. Benign lesion was selected up to two in a patient. We placed VOI (volume of interest) on the tumors and inflammatory lesions based on CT information. Four different PET parameters were measured to compare the two studies: SUV_max_, SUV_mean_ (threshold SUV, 3), average activity over 30 % of the isocontour (isocontour, Bq/mL), and isocontour-mediastinal activity (Bq/mL). Mediastinal activity was measured in the ascending aorta as a standard background activity.

**Fig. 1:**
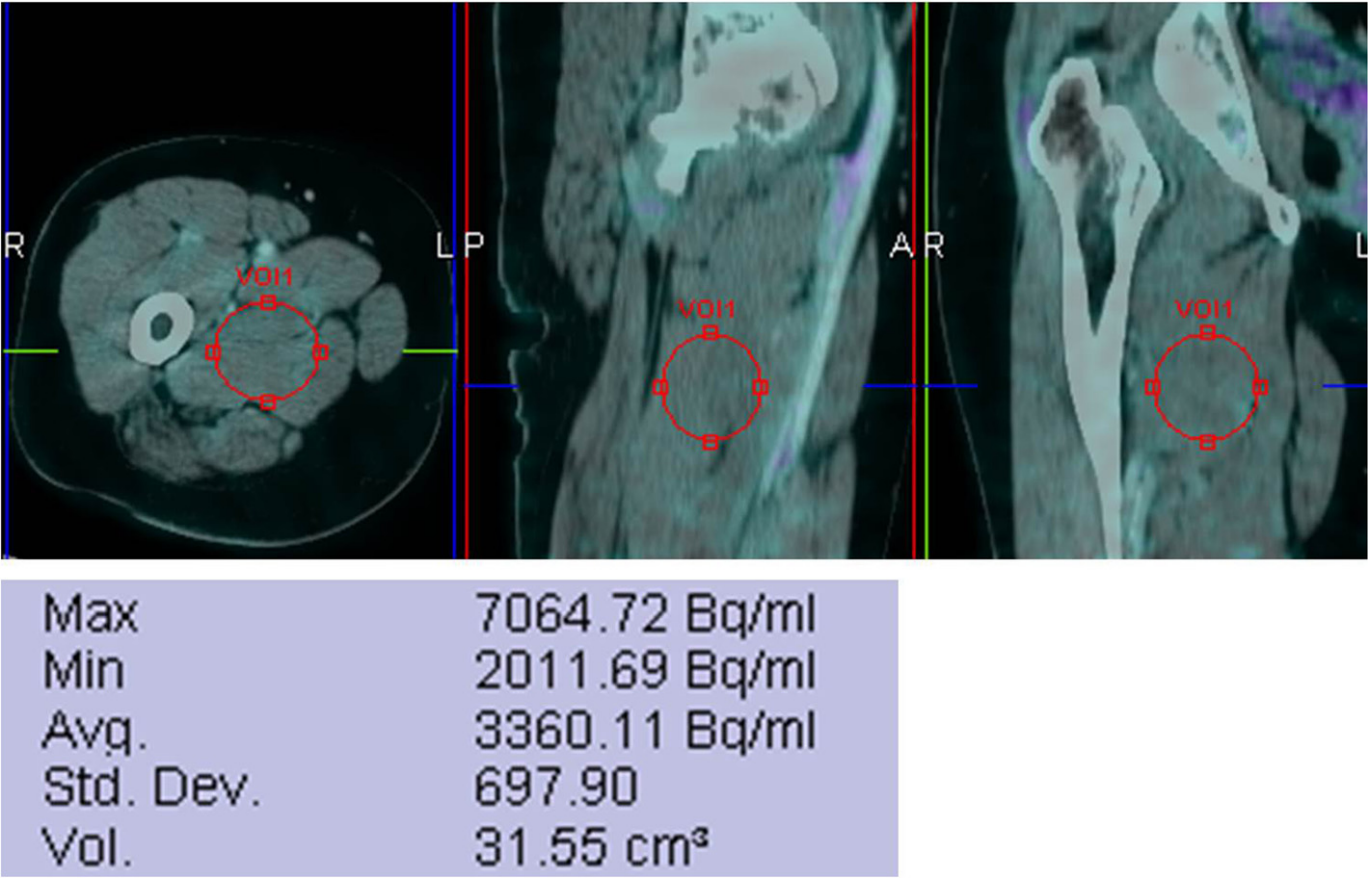
Measurement of the thigh muscle activity. Volume of interest is placed over an area of homogenous activity in the adductor muscles. Average activity in the thigh muscle is 3360.11 Bq/mL

We classified the patients into two groups: the responder or non-responder group according to the presence of PIO effect by measuring thigh muscle uptake. Average activity in thigh muscle was obtained from a volume of interest placed over an area of homogenous activity in the adductor muscles. Care was taken to avoid large vascular structures and any areas of increased ^18^F-FDG uptake that might represent malignancy. The way to measure thigh muscle activity is shown in Fig. 1.

Patients who showed increased activity (average activity measured as Bq/mL) in the thigh muscle after administration of PIO were classified into the responder group. The other patients who showed no change or decreased activity in the thigh muscle were classified into the non-responder group. We calculated the percentage of difference of the PET parameters from the two studies in the same patient. Next, PET parameters of malignant lesions were analyzed based on the type of primary malignancy and were compared with those of inflammatory lesions.

Malignant and inflammatory lesions were enrolled and analyzed by two nuclear physicians. Any equivocal lesions which were confused as tumors or inflammatory lesions were excluded. Because four different PET parameters and mediastinal activity were measured automatically, there were almost no disagreements between two nuclear physicians. Few parameters were different but showed similar values. They were applied as their average values.

### Statistical analysis

Paired *t* test and Wilcoxon signed rank test were used to evaluate the differences in PET parameters in same lesions. Mann-Whitney *U* test was performed for comparing the parameters of malignant and inflammatory lesions. Any lesion which showed difference more than 1 standard deviation was regarded as a meaningful change. Statistical analysis was performed using SPSS software (version 12.0). Statistical significance was defined as a *P* value less than 0.05.

## Results

### Patient characteristics

Forty-three patients from Chonbuk National University Hospital were recruited in this prospective study. Mean age of patients was 64.0 years. There were various types of malignancies: cancers of the hepatobiliary tree and pancreas (except for hepatocellular carcinoma), lung, liver (hepatocellular), colorectum, stomach, esophagus, and kidney. Benign lesions included mediastinal lymph node, pneumonia, rib fracture, and liver cyst. Patient details are summarized in Table 1.

**Table 1:** Characteristics of patients

Characteristics					
Age (mean ± SD, year)	64.0 ± 11.8				
Sex (M:F)	30:13				
Type of malignancy	Responder (lesion/patient)	Non-responder (lesion/patient)	Benign lesion	Responder (lesion/patient)	Non-responder (lesion/patient)
Hepatobiliary and pancreatic cancer	18/9	6/4	Mediastinal lymph node	9/7	4/3
Hepatocellular carcinoma	7/6	5/4	Pneumonia	1/1	–
Colorectal cancer	7/5	2/1	Rib fracture	–	1/1
Gastric cancer	4/4	7/4	Liver cyst	–	1/1
Esophageal cancer	3/1	2/1			
Renal cell carcinoma	2/1	–			
Duodenal GIST	1/1	–			
Lung cancer	–	1/1			

Of the 43 patients, 29 patients who showed increased FDG uptake in the right thigh muscle after administration of PIO were classified into the responder group. The other 14 patients who did not show increased FDG uptake were categorized into the non-responder group. Average activity of the thigh muscle increased from 2346.57 ± 554.26 to 2986.24 ± 689.45 Bq/mL in responder group. In non-responder group, it decreased from 2457.33 ± 386.55 to 2173.23 ± 324.04 Bq/mL.

Myocardial activity was evaluated by measuring average activity in left ventricle. Myocardial activity changed from 8433.25 ± 2816.97 to 8768.34 ± 2184.25 Bq/mL in responder group and from 8941.13 ± 2247.61 to 8845.67 ± 2713.55 Bq/mL in non-responder group. There were no significant differences both when comparing two groups and comparing two different days.

In comparison of fasting blood glucose level and body mass index between responder and non-responder groups, significant differences were not observed. Fasting blood glucose levels in responder group changed from 91.67 ± 16.10 to 89.24 ± 19.31 mg/dL, and those of non-responder group changed from 89.32 ± 18.66 to 84.86 ± 13.98 mg/dL. Body mass index (BMI) was calculated by dividing weight in kilograms by the square of height in meters. They were 23.11 ± 3.06 kg/m^2^ in responder group and 21.71 ± 2.56 kg/m^2^ in non-responder group.

We recommend the dose of PIO two tablets (30 mg) to the patients enrolled; however, some of them did not easily accept the two tablets and wanted to take one tablet (15 mg). Therefore, 13 patients were administered one tablet of PIO (15 mg) and the other 30 patients took two tablets of PIO (30 mg). Most patients showed slightly lower plasma glucose level after administration of PIO. On comparison of laboratory data, there were no clinically adverse changes after administration of PIO.

### Analysis of PET parameters

**Fig. 2:**
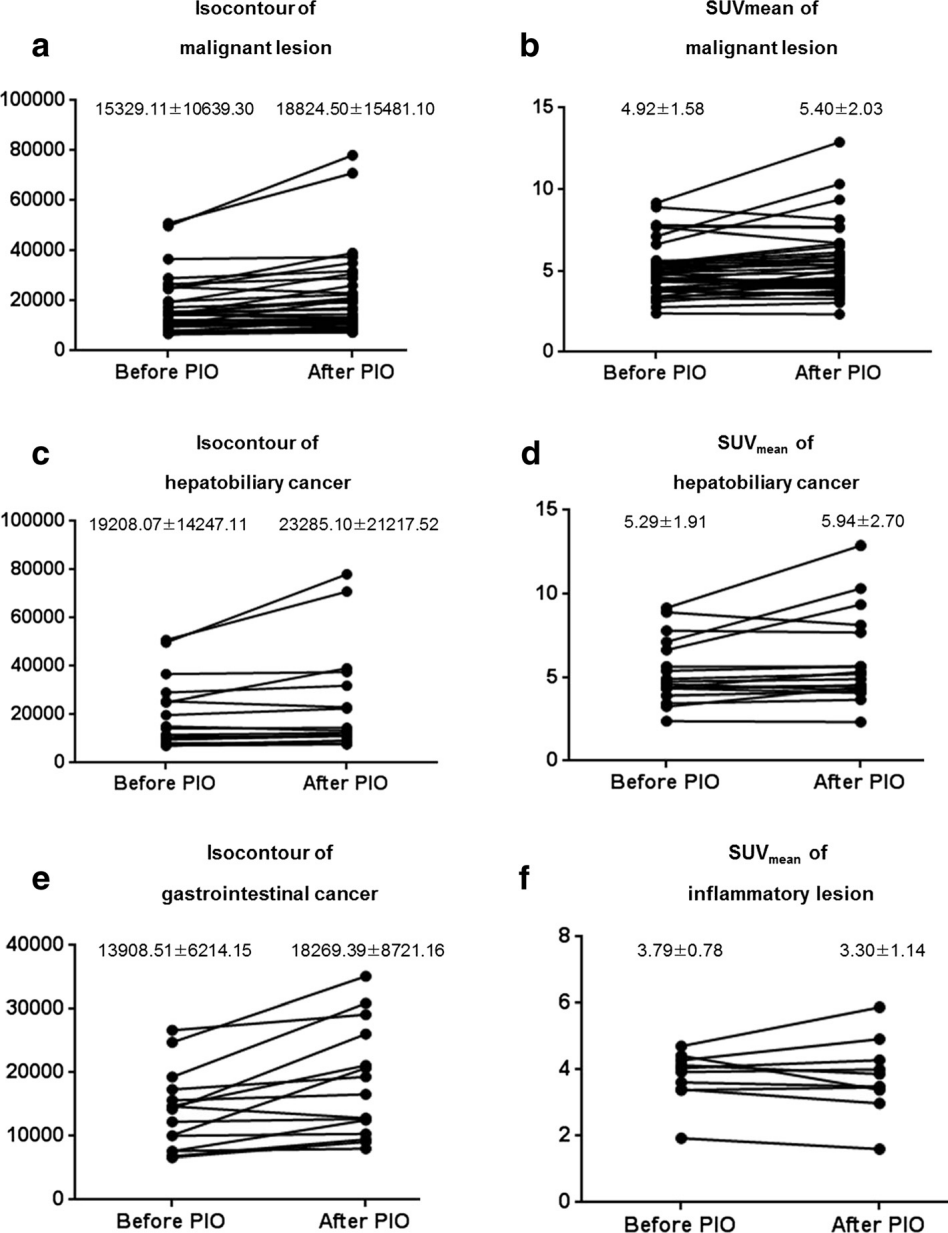
Changing patterns of PET parameters in the responder group. **a**, **b** Isocontour and SUV_mean_ show increasing pattern in malignant lesions after the use of pioglitazone. **c**, **d** In hepatobiliary cancer, 12 out of the 18 lesions show increasing patterns of SUV_mean_. Four out of the 12 increase more than 1 SD. **e** In gastrointestinal cancer, 14 out of the 15 lesions have increasing patterns of isocontour. Six out of the 14 lesions show significant increase more than 1 standard deviation (SD). **f** On the other hand, in inflammatory lesions, there is no statistically significant increase

**Fig. 3:**
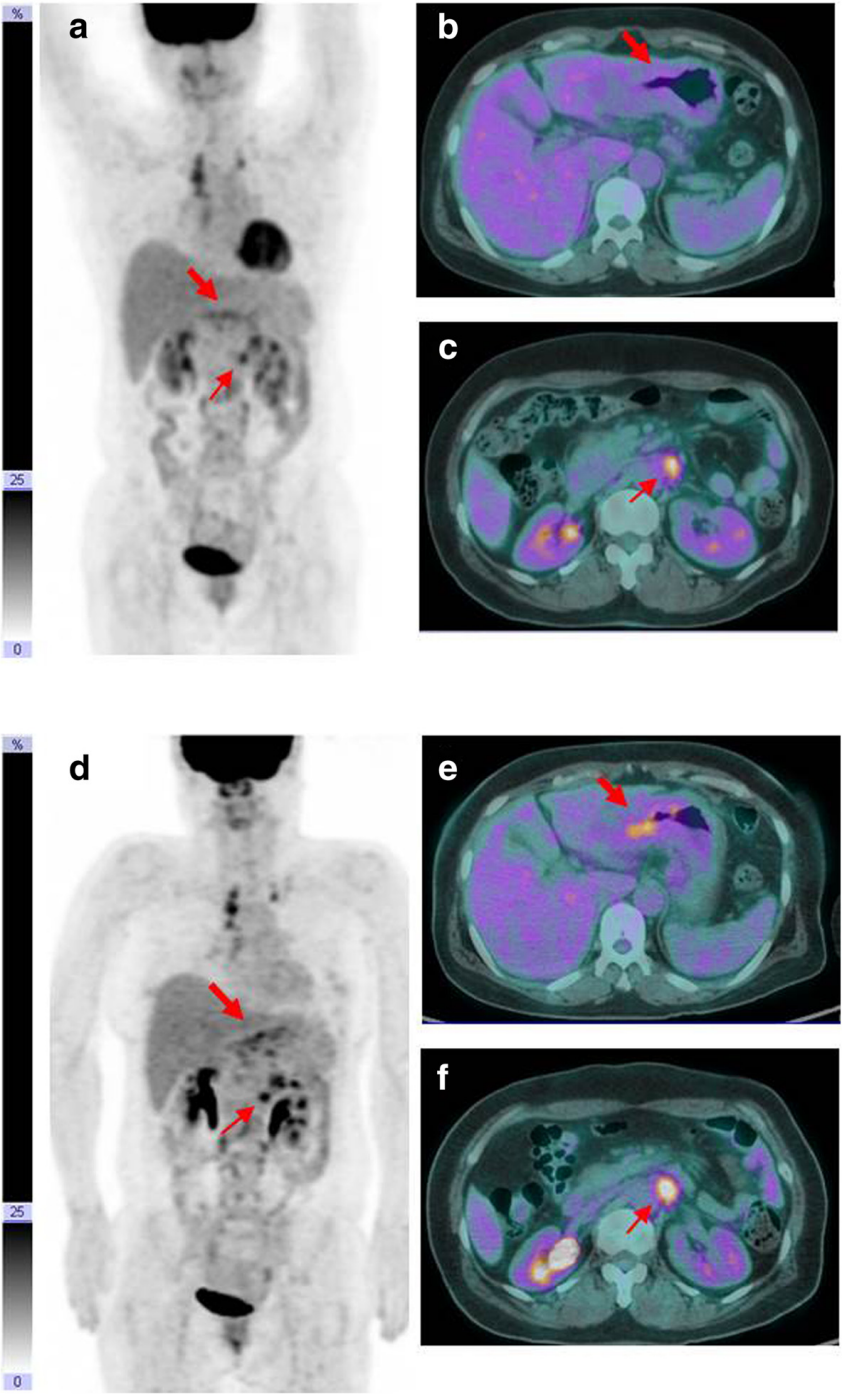
A 52-year-old woman with gastric cancer and multiple lymph nodes metastases. **a**, **b** and **c** Before administration of pioglitazone, SUV_mean_ and isocontour of gastric cancer in ^18^F-FDG PET/CT image were 3.09 and 6824.52 Bq/mL. **d**, **e** and **f** After administration of pioglitazone, they were increased to 3.78 and 9480.92 Bq/mL. In both maximum-intensity-projection and axial PET/CT images, glucose metabolism in gastric cancer and metastatic paraaortic lymph node shows increasing pattern on the next day after the use of pioglitazone. This patient also has both supraclavicular and right mediastinal lymph nodes metastases

Forty-two malignant lesions and 10 benign lesions in the responder group and 23 malignant lesions and 6 benign lesions in the non-responder group were analyzed by using the following four different PET parameters: SUV_max_, SUV_mean_, isocontour, and isocontour-mediastinal activity. All four PET parameters of malignant lesions in the responder group showed increasing patterns after the use of PIO. In the subgroup analysis, the similar patterns were observed in gastrointestinal cancer. In hepatobiliary and pancreatic cancer (except for hepatocellular carcinoma), SUV_mean_ and isocontour showed a statistically significant increase in the presence of PIO. Hepatocellular carcinoma showed increasing patterns of PET parameters, but these were not statistically significant. Statistically useful parameters were different depending on the type of primary malignancy. In inflammatory lesions, SUV_mean_ and isocontour-mediastinal activity showed decreasing patterns and isocontour-mediastinal activity showed the most apparent decrease after the use of PIO. On comparison of the percentage of difference between malignant and inflammatory lesions, isocontour of malignant lesions showed statistically significant differences. This parameter was considered to have differential power to dichotomize lesions into malignancy versus inflammation. The percentage of difference and the changing patterns of PET parameters in the responder group are shown in Table 2 and Fig. 2. Figure 3 is ^18^F-FDG PET/CT images of a patient who have malignant lesions.

**Table 2:** Percentage of difference of PET parameters in responder group

Type of lesions	SUV_max_	SUV_mean_	Isocontour (Bq/mL)	Isocontour-mediastinal activity(Bq/mL)
Malignant lesion	13.82*	9.49*	19.62*^,^**	20.37*
Gastrointestinal cancer	25.94*	9.69	33.19*	41.59*
Hepatobiliary and pancreatic cancer	5.89	10.71*	14.40*	8.73
Hepatocellular carcinoma	7.48	3.98	2.83	5.49
Inflammatory lesion	5.95	−1.27	8.91	−14.99

*P < 0.05 when compared parameters between the first day and second day

**P < 0.05 when compared with difference (%) of inflammatory lesion

**Fig. 4:**
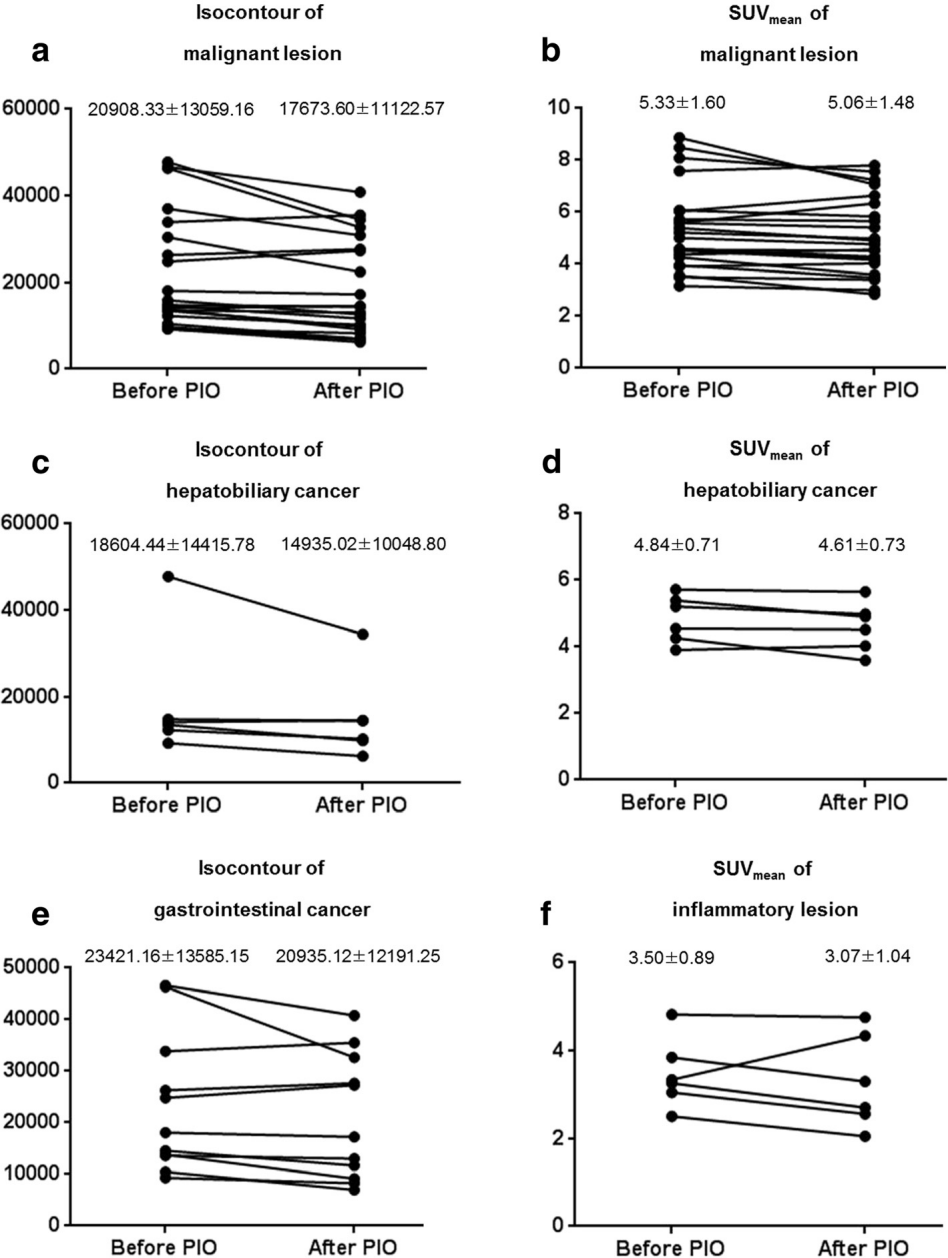
**a**, **b**, **c**, **d**, **e**, **f** Changing patterns of PET parameters in the non-responder group. The PET parameters show decreasing patterns in both malignant and inflammatory lesions after the use of PIO. There are no significant differences

In the non-responder group, all four PET parameters showed decreasing patterns in both malignant and inflammatory lesions after the use of PIO. There was no statistically significant difference in PET parameters of malignant lesions. In inflammatory lesion, isocontour, and isocontour-mediastinal activity showed statistically significant decrease after use of PIO. The % difference and the changing patterns of PET parameters in the non-responder group are shown in Table 3 and Fig. 4.

**Table 3:** Percentage of difference of PET parameters in non-responder group

Type of lesions	SUV_max_	SUV_mean_	Isocontour (Bq/mL)	Isocontour-mediastinal activity (Bq/mL)
Malignant lesion	−4.71	−4.89	−16.36	−12.98
Gastrointestinal cancer	−5.88	−4.68	−11.64	−8.74
Hepatobiliary and pancreatic cancer	−6.71	−4.64	−17.34	−8.48
Hepatocellular carcinoma	−1.64	−5.30	−25.52	−26.99
Inflammatory lesion	−10.21	−6.17	−32.22*	−45.70*

*P < 0.05 when compared parameters between the first day and second day

Contrary to our previous preclinical studies, in comparison of PET parameters between one tablet (15 mg) group and two tablets (30 mg) group, PET parameters did not show dose-dependent manners.

Additionally, in analyzing tumor features on CT such as size and enhancement pattern, there were no specific relationships with four different PET parameters.

## Discussion

The thiazolidinediones or “glitazones” are one class of oral antidiabetic drugs that improve insulin sensitivity in patients with type 2 diabetes [27]. In our previous preclinical study, higher ^18^F-FDG uptake was observed after the injection of rosiglitazone in malignant xenograft mice, but no significant change was observed in inflammatory xenograft mice. We found that rosiglitazone had the potential to increase the contrast between malignant and inflammatory lesions. Some studies have however reported that rosiglitazone is associated with an increased risk of heart attack and hear failure [28, 29]. In 2010, the FDA took a cautious stance and limited exposure to rosiglitazone [30]. The rosiglitazone discontinuation rate increased following the FDA warning, and currently, rosiglitazone is almost pulled out of the market [31]. Therefore, a preclinical study using PIO rather than rosiglitazone was performed to investigate the differential effects on malignant and inflammatory lesions. We also found that ^18^F-FDG uptake was increased in response to PIO treatment in malignant cells in a dose-dependent manner, while ^18^F-FDG uptake was decreased in macrophages. In vivo studies also showed that ^18^F-FDG uptake (%ID/g) in malignant lesions was increased by 1.5-fold and ^18^F-FDG uptake (%ID/g) in inflammatory lesions was decreased by 1.8-fold after treatment with PIO.

Based on these preclinical results, we established the clinical study design to assess the PIO effect in differentiating malignant lesions from inflammatory lesions on ^18^F-FDG PET imaging. Forty-three patients were enrolled in this study and they underwent ^18^F-FDG PET/CT twice in the absence and presence of PIO. We chose the thigh muscle as a reference region to validate the action of PIO. This was because the thigh muscle was considered to be less affected by the patient’s tension or cold environment and wide enough for measuring the average ^18^F-FDG activity. Because PIO improves insulin sensitivity and increases cellular glucose uptake from the blood, the thigh muscle activity on ^18^F-FDG PET imaging should increase after the use of PIO. Twenty-nine patients (67.4 %) showed increased muscular activity, but the other 14 patients (32.6 %) did not show increased muscular activity after treatment with PIO. The findings of 14 non-responder patients could be explained by the fact that all of these patients had a low-fasting blood glucose level <126 mg/dL, and PIO action probably might not be necessary to decrease the blood glucose level by transporting blood glucose into the peripheral tissue in some patients. This could be a possible reason why there were no statistically significant differences in PET parameters in the non-responder group.

Interestingly, there were some patients who showed decreasing patterns of PET parameters in malignant lesions, regardless of the presence of the PIO effect. Generally, malignant tumor tissues include not only cancer cells but also host stromal cells (e.g., fibroblasts, macrophages, lymphocytes, etc.) [32]. ^18^F-FDG uptake could be affected by these stromal cells [33–35] and it might decrease in tumor tissues, which include a large proportion of inflammatory cells, after the use of PIO. However, further investigations are required to elucidate the relationship between intratumoral heterogeneity and PIO effect.

We measured four different PET parameters: SUV_max_, SUV_mean_, average activity over 30 % of the isocontour (isocontour), and isocontour-mediastinal activity. These parameters could be easily measured on a workstation without any expensive or complicated software. All of these four PET parameters were increased in malignant lesions, and the same patterns were observed in the subgroup analysis of gastrointestinal cancer. In the subgroup analysis of hepatobiliary and pancreatic cancer, SUV_mean_ and isocontour showed statistically significant increase after treatment with PIO. These results suggest that the useful PET parameters would be different depending on the type of primary malignancy. A further study in a large number of patients is necessary to establish the specific diagnostic protocol.

The Korean FDA allowed us to administer a PIO dose up to three tablets (45 mg) in each patient. The dosage of PIO was recommended 30 mg to provide same pharmacological condition to the enrolled patients; however, 13 patients wanted to take one tablet (15 mg), and the other 30 patients took two tablets (30 mg) of PIO. In comparison of PET parameters between one tablet group and two tablets group, PET parameters did not show dose-dependent manners. Prolonged use of PIO has been known sometimes to bring several side effects: anemia, hypoglycemia induced symptoms such as palpitation, dizziness, diaphoresis, congestive heart failure, pulmonary edema, and myocardial ischemia. Patients in this study were administered PIO only once, and the maximum dose was 30 mg. None of the patients suffered from adverse effects of PIO. On comparison of laboratory data obtained before and after the study, we could not find any clinically adverse changes after the use of PIO. Based on these results, we can carefully deduce that PIO could be safely applied in the clinical field.

This study has several limitations. Firstly, not all of the lesions were histopathologically confirmed. Primary malignant sites were confirmed by biopsy, but metastatic lymph nodes and metastases in other organs were mostly diagnosed based on serial follow-up. Twenty-four out of 42 malignant lesions and 4 out of 10 benign lesions in responder group and 11 out of 23 malignant lesions and 2 out of 6 benign lesions in non-responder group were confirmed by histopathologic study. The other lesions were determined as malignant or benign by serial follow-up for at least 1 year. The second limitation is the small sample size of benign lesions. Because we could enroll patients who had malignancies, benign lesions were searched in ^18^F-FDG PET/CT images of the same patients. The small sample size of benign lesions might be a possible reason for statistically insignificant values of some important parameters. The third limitation is that lesions in non-responders could not be evaluated as either benign or malignant lesions using these study results. Therefore, a further study in a larger cohort is needed. Further larger-scale study is required to evaluate day-to-day variability of thigh muscle uptake, optimal dose and time of PIO administration, and the effects of other conditions such as oral absorption of PIO, distribution volume, metabolic syndrome, or the severity of the underlying disease.

In spite of these limitations, this study is valuable as it is the first study to investigate PIO effect on ^18^F-FDG uptake in malignant or inflammatory lesions in clinical ^18^F-FDG PET/CT images. In addition, we provide a specific parameter which might be useful to differentiate malignant lesions from inflammatory lesions. We also found that ^18^F-FDG PET technology is a very sensitive tool to show the effect of pharmacologic action on specific target organ. In this study, non-responders who did not show increased ^18^F-FDG uptake in muscle against PIO effect did not show the increased tumor uptake, either. From this point, we think that PET technology can provide us visual and quantitative information whether a drug would work properly or not.

## Conclusions

Although results showed at our preclinical studies had not been drawn in non-responder group and further larger-scale studies are necessary to generalize the potential role of PIO on ^18^F-FDG uptake in all patients, we found that PIO had the potential to increase ^18^F-FDG uptake in malignant lesions in the patients who showed PIO effect on thigh muscle.

## Abbreviations

^18^F-FDG: ^18^F-2-fluoro-2-deoxy-D-glucose
Glut: glucose transporter
Isocontour: average activity over 30 % of the isocontour
PET/CT: positron emission tomography/computed tomography
PIO: pioglitazone
PPAR-γ: peroxisome proliferator activated receptor-γ
